# Supplementary material for: Blastospores from Metarhizium anisopliae and Metarhizium rileyi Are Not Always as Virulent as Conidia Are towards Spodoptera frugiperda Caterpillars and Use Different Infection Mechanisms
Source: Microorganisms. 2023 Jun 16;11(6):1594. doi: 10.3390/microorganisms11061594 (PMC10305327; doi:10.3390/microorganisms11061594)
Supplement: Supplementary file 1 [file microorganisms-11-01594-s001.zip › Supplementary Files_Table S1.pdf]

Table S1. Summary of RNA-Seq read filtering and mapping. Values represent each of the five biological replicates for each treatment. Samples 1 – 10 were mapped with the *Metarhizium anisopliae* reference genome: GCA_013305495.1. Samples 11 – 20 were mapped with the *Metarhizium rileyi* reference genome: GCA_007866325.1.

Sample	Treatment	Number of Input Reads	Uniquely mapped Reads (%)	Reads mapped to too many loci (%)	Unmapped reads (%)
1	*Spodoptera frugiperda* cuticle + *Metarhizium anisopliae* blastospores	17582304	0.14%	0,36%	99,03%
2	*Spodoptera frugiperda* cuticle + *Metarhizium anisopliae* blastospores	15741262	0.15%	0,30%	99,07%
3	*Spodoptera frugiperda* cuticle + *Metarhizium anisopliae* blastospores	14721168	0.10%	0,24%	99,26%
4	*Spodoptera frugiperda* cuticle + *Metarhizium anisopliae* blastospores	17926588	0.15%	0,28%	99,11%
5	*Spodoptera frugiperda* cuticle + *Metarhizium anisopliae* blastospores	13467826	0.14%	0,35%	98,91%
6	*Spodoptera frugiperda* cuticle + *Metarhizium anisopliae* conidia	15694999	0.57%	0,32%	98,66%
7	*Spodoptera frugiperda* cuticle + *Metarhizium anisopliae* conidia	15941969	0.63%	0,55%	98,09%
8	*Spodoptera frugiperda* cuticle + *Metarhizium anisopliae* conidia	16652007	0.36%	0,30%	98,89%
9	*Spodoptera frugiperda* cuticle + *Metarhizium anisopliae* conidia	16245901	0.55%	0,91%	97,35%
10	*Spodoptera frugiperda* cuticle + *Metarhizium anisopliae* conidia	16604615	0.36%	0,65%	98,12%
11	*Spodoptera frugiperda* cuticle + *Metarhizium rileyi* blastospores	17243904	0.32%	0,00%	99,67%
12	*Spodoptera frugiperda* cuticle + *Metarhizium rileyi* blastospores	15728103	0.19%	0,00%	99,80%
13	*Spodoptera frugiperda* cuticle + *Metarhizium rileyi* blastospores	16375249	0.23%	0,00%	99,75%
14	*Spodoptera frugiperda* cuticle + *Metarhizium rileyi* blastospores	15457153	0.24%	0,00%	99,75%
15	*Spodoptera frugiperda* cuticle + *Metarhizium rileyi* blastospores	17595338	0.20%	0,00%	99,79%
16	*Spodoptera frugiperda* cuticle + *Metarhizium rileyi* conidia	17249224	0.49%	0,00%	99,49%
17	*Spodoptera frugiperda* cuticle + *Metarhizium rileyi* conidia	15896404	0.40%	0,00%	99,59%
18	*Spodoptera frugiperda* cuticle + *Metarhizium rileyi* conidia	17481701	0.95%	0,00%	99,04%
19	*Spodoptera frugiperda* cuticle + *Metarhizium rileyi* conidia	14985625	1.12%	0,00%	98,85%
20	*Spodoptera frugiperda* cuticle + *Metarhizium rileyi* conidia	14836469	0.25%	0,00%	99,74%
