# Supplementary material for: Blastospores from Metarhizium anisopliae and Metarhizium rileyi Are Not Always as Virulent as Conidia Are towards Spodoptera frugiperda Caterpillars and Use Different Infection Mechanisms
Source: Microorganisms. 2023 Jun 16;11(6):1594. doi: 10.3390/microorganisms11061594 (PMC10305327; doi:10.3390/microorganisms11061594)
Supplement: Supplementary file 1 [file microorganisms-11-01594-s001.zip › Supplementary Files_Table S2.pdf]

Table S2. Summary of RNA-Seq read filtering and mapping with *Spodoptera frugiperda* reference genome. Values represent each of the five biological replicates for each treatment. All the samples were mapped with the *S. frugiperda* reference genome: GCA_019297735.1

Sample	Treatment	Number of Input Reads	Uniquely mapped Reads (%)	Reads mapped to too many loci (%)	Unmapped reads (%)
1	*Spodoptera frugiperda* cuticle + *Metarhizium anisopliae* blastospores	17546491	68,42%	0,06%	23,59%
2	*Spodoptera frugiperda* cuticle + *Metarhizium anisopliae* blastospores	15707692	66,08%	0,15%	25,91%
3	*Spodoptera frugiperda* cuticle + *Metarhizium anisopliae* blastospores	14700702	66,56%	0,11%	26,32%
4	*Spodoptera frugiperda* cuticle + *Metarhizium anisopliae* blastospores	17887751	66,43%	0,11%	25,96%
5	*Spodoptera frugiperda* cuticle + *Metarhizium anisopliae* blastospores	13441206	67,80%	0,13%	23,98%
6	*Spodoptera frugiperda* cuticle + *Metarhizium anisopliae* conidia	15590528	67,48%	0,05%	25,75%
7	*Spodoptera frugiperda* cuticle + *Metarhizium anisopliae* conidia	15827454	64,15%	0,06%	28,21%
8	*Spodoptera frugiperda* cuticle + *Metarhizium anisopliae* conidia	16581026	62,82%	0,06%	30,52%
9	*Spodoptera frugiperda* cuticle + *Metarhizium anisopliae* conidia	16126915	67,18%	0,06%	21,76%
10	*Spodoptera frugiperda* cuticle + *Metarhizium anisopliae* conidia	16526741	66,13%	0,08%	22,96%
11	*Spodoptera frugiperda* cuticle + *Metarhizium rileyi* blastospores	17187484	68,97%	0,12%	21,31%
12	*Spodoptera frugiperda* cuticle + *Metarhizium rileyi* blastospores	15696812	71,16%	0,15%	21,56%
13	*Spodoptera frugiperda* cuticle + *Metarhizium rileyi* blastospores	16335133	75,19%	0,09%	17,07%
14	*Spodoptera frugiperda* cuticle + *Metarhizium rileyi* blastospores	15418547	72,16%	0,09%	20,43%
15	*Spodoptera frugiperda* cuticle + *Metarhizium rileyi* blastospores	17557909	70,08%	0,15%	20,43%
16	*Spodoptera frugiperda* cuticle + *Metarhizium rileyi* conidia	17161999	68,81%	0,09%	24,35%
17	*Spodoptera frugiperda* cuticle + *Metarhizium rileyi* conidia	15831745	70,15%	0,13%	20,28%
18	*Spodoptera frugiperda* cuticle + *Metarhizium rileyi* conidia	17313507	66,22%	0,10%	23,39%
19	*Spodoptera frugiperda* cuticle + *Metarhizium rileyi* conidia	14814037	68,07%	0,12%	21,03%
20	*Spodoptera frugiperda* cuticle + *Metarhizium rileyi* conidia	14797306	72,48%	0,13%	21,25%
