# Supplementary material for: Blastospores from Metarhizium anisopliae and Metarhizium rileyi Are Not Always as Virulent as Conidia Are towards Spodoptera frugiperda Caterpillars and Use Different Infection Mechanisms
Source: Microorganisms. 2023 Jun 16;11(6):1594. doi: 10.3390/microorganisms11061594 (PMC10305327; doi:10.3390/microorganisms11061594)
Supplement: Supplementary file 1 [file microorganisms-11-01594-s001.zip › Supplementary Files_Table S3.pdf]

Table S3. Up-regulated Protein families and their general functions expressed in *Metarhizium anisopliae* and *Metarhizium rileyi* propagules during penetration on *Spodoptera frugiperda* cuticle.

	Total	PFAMs	Protein Name	Function
**Shared by all structures**	1	PF00067	Cytochrome P450 4F5	Oxidative stress
**Shared by *M. anisopliae* blastospores and conidia**	1	PF00172	Zn(2)-C6 fungal-type DNA-binding domain	Cell function
**Shared by *M. rileyi* blastospores and conidia**	3	PF00155	Aminotransferase, class I/classII	Oxidative stress
		PF13499	EF-hand domain	Virulence factor
		PF13641	Glycosyltransferase like family 2	Oxidative stress
**Shared by *M. anisopliae* and *M. rileyi* blastospores**	1	PF00011	Heat shock protein	Heat shock
**Shared by *M. anisopliae* and *M. rileyi* conidia**	19	PF00171	Aldehyde dehydrogenase domain	Virulence factor
		PF01198	Ribosomal protein L31e	Cell function
		PF02629	CoA-binding	Virulence factor
		PF00903	Glyoxalase/fosfomycin resistance/dioxygenase do	Virulence factor
		PF11327	Egh16-like virulence factor	Virulence factor
		PF01775	Ribosomal protein 50S-L18Ae/60S-L20/60S-L18A	Cell function
		PF01425	Amidase signature domain	Virulence factor
		PF02353	Mycolic acid cyclopropane synthetase	Virulence factor
		PF01042	Endoribonuclease L-PSP	Cell function
		PF00096	Zinc finger C2H2-type	Cell function
		PF01384	Phosphate transporter	Nutrient and substance transport
		PF05368	NmrA-like domain	Cell function
		PF13193	AMP-binding enzyme, C-terminal domain	Virulence factor
		PF00125	Histone H2A/H2B/H3	Virulence factor
		PF00153	Mitochondrial substrate/solute carrier	Cell function
		PF07732	Multicopper oxidase, N-termianl	Virulence factor
		PF03576	Peptidase S58, DmpA	Virulence factor
		PF02136	Nuclear transport factor 2	Nutrient and substance transport
		PF13561	Enoyl-(Acyl carrier protein) reductase	Cell function
